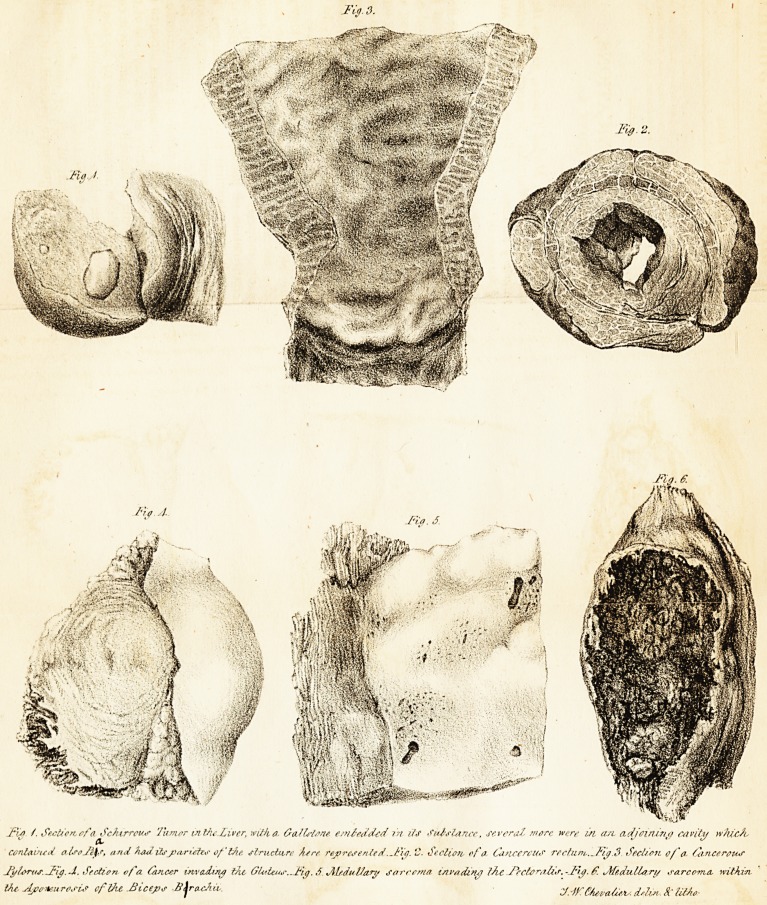# General Observations on Morbid Tumors, and on the Different Modes in Which the Muscular Structure Is Attacked by Cancer and Medullary Sarcoma

**Published:** 1826-02

**Authors:** Thomas Wm. Chevalier

**Affiliations:** Consulting Surgeon to the Royal Union Association.


Fig. 1. Section of a Schirrous Tumor in the Liver, with a Gallstone embedded in its substance, several more were in an adjoining cavity which contained also Peas, and had its parietes of the structure here represented._Fig. 2. Section of a Cancerous rectum._Fig. 3. Section of a Cancerous Pylorus. Fig. 4. Section of a Cancer invading the Gluteus._Fig. 5. Medullary sarcoma invading the Pectoralis._Fig. 6. Medullary sarcoma within the Aponeurosis of the Biceps Brachii.

J. W. Chevalier. delin. & litho.

THE LONDON
### Medical and Physical Journal.

Nº 2 OF VOL. LV.] FEBRUARY, 1826. [Nº 324.

For many fortunate discoveries in medicine, and for the detection of numerous errors, the world is indebted to the rapid circulation of Monthly Journals; and there never existed any work, to which the Faculty, in Europe and America, were under deeper obligations, than to the Medical and Physical Journal of London, now forming a long, but an invaluable, series.—RUSH.

### ORIGINAL COMMUNICATIONS,
### SELECT OBSERVATIONS, &c.

ART. I.—*General Observations on Morbid Tumors, and on the different Modes in which the Muscular Structure is attacked by Cancer and Medullary Sarcoma.* By THOMAS WM. CHEVALIER, Esq. Consulting Surgeon to the Royal Union Association.

[With an Engraving.]

ONE principal object of this paper is to suggest, for the consideration of the reader, how far the ancient doctrine of final causes may be applied to the classification of disease, and especially to that of morbid tumors. For, from the following view of the progress of those two diseases more particularly considered here, it would appear that that doctrine leads to an easier method of learning and distinguishing them, in their early stages, and in their general characters as described, than can be obtained by paying attention merely, or principally, to the phenomena which either of them successively exhibits.

In the other object which the author had chiefly in view, he is sincerely happy to find that he has been to some extent anticipated by the accurate observations of Mr. Wardrop, which have already induced that gentleman to say, with every requisite confirmation of his words annexed, that "he thinks there are sufficient grounds to consider fungus hæmatodes as a morbid change of structure specifically distinct, and in every point of view, different from cancer." It is, therefore, hoped that an additional proof of the rectitude of that gentleman's opinions concerning two maladies hitherto incurable, will not be considered unworthy of the attention of the medical world, nor unwelcome to himself; especially as it consists in a description (which the author believes to be new) of the totally different methods in which one of the essential animal structures is invaded by those two diseases.

Every ultimate produce or effect, in art or nature, implies that some certain specific changes have preceded it, as its cause; and every intermediate change (that is fully comprehended) denotes that some one individual effect will be produced as its immediate result.

In disease, also, we know the intermediate changes that shall succeed, and the ultimate effect that shall result, from the proper insertion of vaccine, or of variolous, or of syphilitic poison, beneath the rete mucosum; and we know that an incapacity for the small-pox, connected with the appearance of a certain kind of cicatrix, is a proof that certain intermediate changes have been passed through.

It is by that which we know would be the result, that we denominate and distinguish diseases, even in their unfinished, or even in their incipient stages; and when any disease hath attained its perfect form, or acmé, it is its perfect form which determines our opinion of its nature.

The acmé of variola, for example, or its ultimate effect on the constitution, is that to which all its proper progress tends; and for the sake of which inoculation was performed. The termination of cancer is that to which all its stages, from the earliest to the latest, are an approach; not only in reference to time, but also in the kind of action in which they severally contist. And, in short, there is not a disease affecting the animal body which is not to be defined either as a progress or a disposition towards the accomplishment, under certain circumstances, of some specific morbid action; or towards the secretion, under certain circumstances, of some specific animal poison; or towards the produce, under certain circumstances, of some specific growth, "*præter, vel contra naturam.*"

I believe it may be asserted generally, that all tumors which do not tend, from any thing essential to their complete form, to implicate surrounding structures in their own morbid characters, consist of substances analogous to some or other of those naturally existing in the body. Mr. Abernethy has observed of some, that "they have no distinguishable peculiarity;" of others, that "their substance, in colour, texture, and size, resembles the larger masses which compose the pancreas;" and I may add, that even the contents of cysts resemble the blood, or the marrow, the sebaceous or mucous secretions, or that which becomes hair, or (as in some partially ossified cysts in the liver and spleen) the substance which unquestionably is the essence of bone.

Hydatids alone are a tribe of preternatural growths, to the structure of which I can find nothing analogous in the natural and healthy body.

A tumor of these kinds, distinguished as merely *præter naturam*, is never found to engage in its own specific mode of growth, nor to retract into its own substance, nor in any way to metamorphose or obliterate neighbouring natural parts. It does not contaminate on its approach, though its disposition to grow, and its actual increase, cannot perhaps be checked or prevented.

From the ill consequences of the presence and growth of such a tumor, the muscles, although sometimes expanded into a web of fasciculi,—the arteries and veins, although sometimes distended and tortuous,—and the nerves, although protruded from their usual courses, may rapidly recover: and hence it may be asserted, that the tendency in the nutritious arteries of these swellings to form and to augment them to a certain indefinite extent, appears all that they have of a morbid character.

The growth of a cancerous tumor is frequently less rapid than that of many comparatively innocuous; but it is always more irresistible, and less evidently modified for the accommodation of organs in its vicinity. A cancer will form inconvenient attachments before it has induced inflammatory action. It disgregates the fasciculi of muscles, and even of nerves, more readily, and with more severe symptoms, than a merely preternatural tumor.

The ultimate tendency of a cancerous tumor is not merely to enlarge, but to ulcerate. The structure of a cancerous tumor, if it resemble any ordinary structure, is most like that of the cellular or parenchymatous substances of the body, when either of them is filled and consolidated by the effusion of coagulated lymph in its interstices; or, in other words, when either of them is prepared for ulceration: and, indeed, whatever tumors are not at all disposed to ulcerate, are by common consent distinguished as not cancerous.

What are called *tubercles of the uterus*, for example, (which present, on their cut surface, an appearance not unlike that of the unimpregnated womb itself,) are easily made to exhibit the striæ commonly enough mentioned as a characteristic of the cut surface of a cancerous growth; but, as these tubercles never ulcerate, no one has ever ventured, so far as I know, to include them among the forms of cancer: and, indeed, they are not like the cancerous structure when minutely examined; for we may always see, in the interstices of the striæ of the section of a cancerous growth, minute portions of a peculiar gristly substance interposed; and this is not to be detected in the section of the tubercle of the uterus.

This interposed substance is like lymph, sufficiently hardened to be both soft and brittle: it has been noticed by several authors; and it would appear to be the means whereby a natural structure is prepared for ulceration. I have seen it in every interstice of a schirrous thyroid gland; and I have known, in two instances, the iliacus internus muscle entirely changed into this or a very similar substance, in patients who died of cancer of the intestinum rectum.*

This interposed substance consolidating the striæ, and not the simple presence of the striæ on a cancerous section, would therefore seem to be the characteristic of the disease; especially as it is observable no where in the natural texture of the body, nor to be any where found in the healthy patient, unless in a surface obviously prepared for ulceration, or in the condensed and ulcerating texture which often surrounds extraneous bodies lodged in the human frame, (*see Plate, fig.* 1:) and hence, indeed, a powerful argument that cancer is, from the very first, a preparative process for malignant ulceration.

In cancerous intestine or stomach, the interposed substance is more abundant than it is in carcinomatous tumors: it is seen composing a much larger proportion of the bulk of the disease (than the striæ or fibrous part composes,) in figures 2 and 3 of the Plate.

When cancer, or any other essentially malignant growth, has once commenced its originally destined and destructive mode of action, it becomes a much more virulent contagion in the neighbourhood of those parts among which it is situated, or even infects the whole body with its own original and potential essence: namely, a certain specific disposition in the arteries to form its substance. It would appear, however, so far as the author has observed, that cancer never becomes so malignant as fungus hæmatodes in general proves itself to have been. I have seen a preparation of a cancerous tumor which has obliterated every trace of the carotid artery: a considerable nerve, however, passing directly through it, is thickened, but not otherwise altered, as far as can be seen; and, indeed, it is a rare occurrence for cancer to obliterate a large artery, and by no means common for it to attack the muscles: whereas, I have repeatedly seen the muscles attacked by fungus hæmatodes; and I have a preparation in my possession of a medullary sarcoma, which has all but opened into the vena cava ascendans, so that the pulpy matter is separated from the cavity of the vein merely by an exceedingly fine membrane; and the aorta is corrugated on the other side of the tumor.

The two chief characteristic distinctions between these two kinds of malignant tumors, do not consist, however, in their possessing a different *degree* of malignancy, or of any other morbid character; nor even in any difference of proper structure, how apparent soever; but in their having appropriated to

* In both these instances, the iliacus internus muscle was changed into an homogeneous pulpy mass, preserving the external form of the muscle, and having no immediate connexion with the disease of the intestine: it had, however, in both instances corroded the ala ilii, and in points begun to ulcerate.

them two distinct terminations, and in their attacking the muscular structure in two distinct ways.

It has been remarked already that cancer terminates in ulceration; and, indeed, wherever the disease arrives at a stage which may be properly called its ultimate result, or acmè, it always does so. If life be cut short before ulceration commences, the disease in that case has not properly terminated: it is found in a progressive and incomplete state; its history, in such cases, is unfinished.

Fungus hæmatodes, on the contrary, if allowed to attain its proper result, invariably terminates, not in ulceration, but in sphacelus.

Cancer may slough from the violence of the ulcerative action, or from the rapidity of its ravages: it never sphacelates, however; for, if I rightly comprehend the term sphacelus, it signifies a failure and cessation of the *simple power to live:* whereas, any other kind of mortification implies that there has been an effort to accomplish more than the mere support of life,—*e. g.* inflammation or ulceration. Again, it is notorious that the skin over a fungus hæmatodes almost always ulcerates; probably from the powers of life in the part yielding before they are sufficiently impaired to be capable of being suddenly arrested like the life of the substance of the tumor, which, as hath been said, invariably sphacelates, unless where the patient's death has cut short the disease in its progress, and before its history is complete.*

In an Essay upon the Pathology of the Muscles, which I submitted to the council of the College of Surgeons, and to which they did me the honour to award the Jacksonian prize, in the year 1822, I gave the following account of the different ways in which I have seen the muscular structure invaded by cancer and medullary sarcoma.

I have twice or thrice observed a cancerous tumor encroaching upon the muscles with which it was in contact, and obliterating the muscular fibres; but in no case more distinctly than in that which first led me to a knowledge of the fact, and which is illustrated by the preparation in my own collection. This preparation consists of a section of a cancerous tumor situated between the integuments and the gluteus maximus of a labouring man. *(See fig. 4.)* The white cartilaginous and lobulated substance composing the bulk of the tumor has all the characters of cancer; the striæ and the interposed substance being both ob-

---

* The blood-vessels of the integuments opened by ulceration pour out blood, as well as those of the substance of the tumor, opened by sphacelation, or burst for want of tone. It may be mentioned here, that all consideration of cancerous ulcers, primarily such, has been here entirely omitted; this paper being merely on Tumors.

servable in its section, and an appearance of incipient ulceration having been noticed at one point (as not uncommonly in true cancer,) in the centre of the schirrous mass. At its upper part, this tumor is represented distinct from the fat; but (as is usual in such cases) it is more and more confused with the surrounding laminæ of cellular membrane, as we approach its centre; so that the circumference of the tumor appears partially composed of constricted cellular substance, and not so pale as its central portion, which is also less membranous, consisting of a larger proportion of the interposed substance, and being indistinctly circumscribed and separated from the rest of the mass of the tumor by a thin lamina of cellular membrane. In the lower part, the cancerous growth is in contact with the coarse fibres of the gluteus muscle; and at this part are to be observed little gristly points and projections, sprouting from the cancerous mass, and insinuating themselves between the muscular fasciculi. Tough shreds of cellular membrane are attached at this part to the tumor, and the muscular fasciculi suddenly terminate in between the bases of the gristly cancerous points before mentioned. It is as though the cancerous growth sprouting between the muscular fasciculi had gradually retracted them into its substance, where all traces of their fibrous or muscular character are suddenly lost, in consequence of the pressure, or at all events of absorption; so that they are, in effect, *inserted* into the tumor; while they remain unaltered without it.

When a tumor of medullary sarcoma forms under a muscle (*e. g.* under the pectoralis major), it becomes attached to the fibres, oftener than cancer appears to do; but it does not adhere so firmly. At first the substance of the fungus is separated from the cords of muscular fibres, by laminæ of cellular membrane interposed; and the distinction between the lobulated surface of the diseased mass and the fasciculi distended over it, is rendered distinct by the homogeneous texture and uniform paleness of the pulpy body, and by the redness (or, in an old preparation, by the brown colour,) of the bundles of fibres. The muscular fasciculi are now continued from beyond the neighbourhood of the disease, across it, and onward to their natural insertion in their tendon; and they are stout enough to admit of being torn up from the surface of the tumor.

After a time, not only does the distinction by the laminæ interposed become less demonstrable, but also the fibres composing the muscular fasciculi have become paler and softer, and more liable to break short off if raised up, and less distinct, one from the rest.

The muscular fibres become less and less distinguishable from one another as the disease continues to advance, and less distinguishable also from the substance of the tumor; and they are rendered so soft that they cannot be torn off. A magnifying glass, however, enables us still to trace the lowest muscular fasciculi from their natural origin, where they are apparently sound, into the substance of the tumor, where they are lost in the homogeneous and uniform white pulp. In the fasciculi above those that, being the most internal, are first lost in the tumor, the disease has not yet so thoroughly exerted its influence: in these one may observe more of the form of muscular fibres, collected in fasciculi; and they may even be traced, by the aid of the glass, right through the diseased mass; though to the naked eye they seem a portion of the medullary sarcoma, in no respect different from its original pale substance. The change is gradual from the homogeneous and uniform pulp composing the central part of the tumor, through successive strata of muscular fibres, all of one magnitude, individually, but in consistence and colour less and less resembling the pulp as we recede from it, until they are found running across it, in a condition to all appearance nearly, or perfectly, healthy. In some examples, the softened and whitened mass of lower fibres is separated from the sounder muscular structure above it, by a membrane apparently the same as that which separates one mass of fasciculi in a healthy muscle from the rest. The invasion of the muscular structure by the medullary sarcoma is not, therefore, to be defined an obliteration of them; but (if I may be allowed the expression,) as a gradual metamorphosis of their proper texture into that of the morbid pulp.* *(See fig. 5.)*

I am not aware that cancer ever begins in the substance of a muscle; but I have several times seen what Mr. Abernethy remarks,—viz. "In the advanced stage of carcinoma, a number of small tumors, of similar structure to the original disease, forming at some distance around it;" and, in the case of E. B., a patient who died in St. George's Hospital some years ago, with cancer in every structure of her chest, except the heart, many little pisiform tumors were found interspersed loose among the fasciculi of the humoral muscles. These small tumors were, however, invested only by the cellular membrane, and no where incorporated with the proper muscular fibre.

The following is a case of medullary sarcoma, beginning within the biceps flexor cubiti; for which, as well as for the preparation, I am indebted to my late father. *(See fig. 6.)*

I. K., in January, many years ago, took the advice of a surgeon of eminence and of distinguished talent, for herpes on his leg. He had also a large tumor on his left arm, containing

---

* It is not asserted that the above are the only ways in which the muscles are invaded by cancer and fungus hæmatodes; although they are all with which the author is acquainted.

a fluid, and evidently connected to the biceps muscle. He attributed this to a strain in the month of June previous. In the beginning of March, the swelling had increased; and, on the 12th, a seton was passed through it, and a small quantity of bloody fluid was discharged. On the next day he had a rigor, and appropriate medicines were prescribed. On the 14th, the febrile symptoms were increased in severity: he had been delirious, and there was now no discharge; but, on the next day, much bloody serum drained away, and the tumor was diminished in size. He had dyspnœa, and expectorated; the pulse was irregular, but not weak. On the 17th, his arm was very sore, the tumor rather less; the pulse still intermitting, but not frequent. On the 18th, he died.

On dissection, the whole tumor was found comprehended in the dilated aponeurosis of the biceps muscle: it contained a large quantity of bloody serum, and its anterior part was in a state of sphacelus; the posterior being that which is represented in figure 6. In the drawing the muscle is portrayed, as it is in the preparation, suspended by its tendon; and the disease appears situated in that portion of its belly which corresponds with the short head, the fleshy part of the long head hanging behind, and on the right of the drawing. The diseased surface is composed of jagged nodules, of medullary sarcoma, mingled with shreds of cellular membrane.

The muscular fibres cannot be traced into the fungous surface in that regular and distinct gradation which is observable in figure 2; but those nodules of medullary consistence and colour, when viewed through the magnifying glass, are seen to consist of muscular fibres, unaltered as to form individually, but shrunk and *resserrées*, like those of a piece of meat on the burning coals. If the points of two needles be introduced, (as in the drawing,) with their points in contact, into one of the nodules of medullary matter, and there gently separated, the substance decidedly splits, and this only in one direction; and, care being taken not to break the softened texture, the glass shows the surface thus torn to be indisputably fibrous, and in every respect the texture of *medullized muscle*, (if such a term may be allowed.)

The change, however, in the length of the fibres, from their natural consistence, tenacity, and colour, to the state of disease implied, is too sudden to allow of their being satisfactorily traced; and it is to be remarked, lastly, that the investing membrane of the fibres and fasciculi remains, in general, in the form of tough and strong shreds, in the midst of the fasciculi changed into the medullary pulp.

Cancer and fungus hæmatodes having been thus shown to be specifically distinct, first in their proper terminations, and secondly in their modes of invading the muscular structure, the nosological question concerning their identity is so far determined in the negative; for, surely, although their dissimilar structures should be found in one and the same tumor, and much more if only in the same subject, there are still sufficient grounds to authorise us to say that such a case is not the exhibition of two concomitant varieties of one disease, but the absolute concomitance of two. And, although the predisposition to either of these diseases should be proved to be one and the same, and the constitutional tendency to both identical in kind, or even in degree, still must they be considered as distinct, if not as nearly opposite, productions.

In regard to the latter of these two diseases, I may add to what I have written, that I have seen the medullary sarcoma, and the mottled variety of fungus hæmatodes, composing different parts of the same tumor. I have also examples of medullary sarcoma, coexistent with the substance of one of those very rare tumors, which consist of a diaphanous and lobulated vascular texture, as soft almost as size, and not very unlike it in appearance; and, with regard to general form and disposition, to be best described as *soft cancer*. Lastly, I have observed all degrees of resemblance and approximation, as well as the accident of coexistence, between the mottled variety and the soft brown or black substance, which, mixed with recent coagula, or not so, is often to be found coexistent with medullary sarcoma.

These four varieties of disease, viz. the medullary sarcoma, the soft cancer-like substance, the mottled variety, and that which is brown or black, I am, therefore, inclined to include in the term fungus hæmatodes; not so much, however, because they are concomitant, as from their having the same history,—the same inevitable disposition ultimately to sphacelate,—the same hæmorrhagic tendency, &c. &c.

*20, South Audley-street; January 2d, 1826.*

## Figures and Tables

**Fig. 1. Fig. 3. Fig. 2. Fig. 4. Fig. 5. Fig. 6. f1:**